# Comparison of androgen receptor mutation detection between plasma extracellular vesicle DNA and cell-free DNA and its relationship to prostate cancer prognosis

**DOI:** 10.1080/07853890.2024.2426770

**Published:** 2024-11-13

**Authors:** Ting Ding, Xiao Li, Longlong Zhang, Zhen Wei, Chaoliang Xiong, Hong Wang, Xiaoke Hao, Xianfei Zeng

**Affiliations:** a School of Medicine, Northwest University, Xi’an, China; b Department of Clinical Laboratory, The Second Affiliated Hospital of Xi’an Jiaotong University, Xi’an, China; c Department of Clinical Laboratory, Xijing Hospital, Fourth Military Medical University (Air Force Medical University), Xi’an, China; d Department of Urology, Xijing Hospital, Fourth Military Medical University (Air Force Medical University), Xi’an, China; e Xi’an Area Medical Laboratory Center, Xi’an, China; f Department of Urology, The Second Affiliated Hospital of Xi’an Jiaotong University, Xi’an, China

**Keywords:** Liquid biopsy, extracellular vesicle DNA, cell-free DNA, AR mutations, prostate cancer

## Abstract

**Background:**

In liquid biopsy, mutation detection is primarily performed using cell-free DNA (cfDNA). However, the numerous advantages of extracellular vesicle (EV) DNA for mutation detection have gradually garnered the attention of researchers in recent years. This study aimed to compare the differences between EV DNA and cfDNA in mutation detection and explore the role of plasma androgen receptor (AR) mutations in the prognosis of prostate cancer (PCa).

**Methods:**

We compared the biological characteristics of plasma extracellular vesicle DNA (p-EV DNA) and cfDNA by capillary electrophoresis and concentration detection. Subsequently, we performed pan-oncogene-targeted sequencing in paired tissue and plasma samples from five patients with PCa to verify the feasibility of mutation detection using p-EV DNA and cfDNA. Further, we conducted AR mutation detection in expanded samples to compare the differences between EV DNA and cfDNA in mutation detection and to analyse their role in PCa.

**Results:**

p-EV DNA fragments were larger than plasma cell-free DNA (p-cfDNA) fragments; however, there was no significant difference in their concentrations in the plasma of patients with PCa. Feasibility analysis revealed that major mutations associated with PCa detected in tissue samples could be identified in both p-EV DNA and p-cfDNA. Advantage comparison found that, although cfDNA could detect more mutations, AR mutations in EV DNA were more strongly associated with a poor prognosis of PCa than cfDNA.

**Conclusion:**

Mutation detection using either EV DNA or cfDNA is both feasible in PCa liquid biopsies, and EV DNA AR mutations have an advantage in prognostic assessment for PCa. This study lays the foundation for future research on EV DNA-related biomarkers.

## 1. Introduction

Prostate cancer (PCa) is one of the most common malignant tumours in men worldwide, with a mortality rate of up to 11% [1]. In the mid-twentieth century, the treatment for prostate cancer was limited to surgical resection and radiation therapy. However, with a further understanding of the biology of prostate cancer, the important role of androgens in the occurrence and progression of prostate cancer is gradually being recognized [2,3]. Androgen receptor (AR) is a protein that regulates the expression of related genes by binding to androgens [4]. It regulates normal physiological processes in normal prostate tissue but is overactivated in prostate cancer and is closely related to its occurrence and progression [3]. Therefore, as a clinically proven mainstream target, AR function inhibition has become an important strategy for prostate cancer treatment [5,6]. Although AR-targeted drugs have shown significant efficacy in PCa treatment, intrinsic and acquired resistance to androgen receptor pathway inhibitor (ARPI) is a major problem in this treatment, which is closely associated with poor prognosis in patients with PCa [7,8]. ARPI resistance can be generated through various mechanisms, among which AR gene mutations are one of the most common [7,9]. Somatic hotspot mutations related to the effectiveness of clinical treatment are mainly concentrated in the ligand-binding domains of AR, including L702H, W742L/C, H875Y, F877L, T878A/S and so on. The detection of AR variations is helpful for guiding medication and predicting the prognosis of patients with PCa [8,10].

Due to the heterogeneity and invasive nature of tissue biopsy, liquid biopsy has become a promising new technology for tumour detection [11]. Currently, several research teams have detected AR variations through cell-free DNA (cfDNA) in patients with PCa and confirmed their association with poor patient prognosis [12,13]. However, using cfDNA for liquid biopsies also has certain limitations: on one hand, cfDNA testing may be prone to false negatives, especially in early tumours, due to the background presence of large amounts of free nucleic acids from normal cells [14]. On the other hand, due to cloned haematopoiesis, cfDNA testing is also prone to produce false-positive results [15]. Extracellular vesicle (EV)-derived DNA has gradually attracted attention as a novel detection target. EV DNA primarily consists of double-stranded DNA, which can reflect the state of the source genomic DNA (gDNA) [16]. Tumour-specific EV DNA can be extracted by isolating tumour-derived EV using the immunoaffinity method [17]. Therefore, EV DNA is anticipated to surpass cfDNA as a superior candidate target for liquid tumour biopsy. So, is EV DNA a better choice for detecting AR mutations? Although previous studies have compared the mutation detection capabilities of EV DNA and cfDNA in a small number of samples [18–20], it remains unclear which sample type holds a comparative advantage.

The aim of this study was to investigate whether tumour-derived AR mutations in the plasma of patients with PCa are primarily present in EV DNA or cfDNA and to determine which target exhibits higher sensitivity in detecting AR mutations. Additionally, we aimed to analyse the relationship between AR mutations found in plasma extracellular vesicle DNA (p-EV DNA) and plasma cell-free DNA (p-cfDNA) and clinical outcomes in patients with PCa.

## 2. Methods

### 2.1. Patients

With informed consent from the patients and approval from the hospital’s ethics committee (No. KY20222066-C-1), we recruited 66 patients with PCa from January to August 2023. These patients were histologically confirmed to have no other concurrent malignancies and were intended to undergo treatment with AR pathway-related drugs at the Department of Urology, the First Affiliated Hospital of the Air Force Medical University. After enrolment, clinical samples were collected from the patients. Among them, 29 patients were collected paired tissue and plasma samples, while the remaining patients were only collected plasma samples. The staging and risk stratification of patients with PC in this study followed the classification outlined in the ‘the European Society for Medical Oncology (ESMO) Clinical Practice Guidelines for Prostate Cancer: Diagnosis, Treatment, and Follow-Up’ and the 8th edition of the American Joint Committee on Cancer (AJCC) Cancer Staging Manual [21,22]. During the follow-up period, we defined disease progression as follows: (1) identification of new metastases or an increase in the volume of primary tumours or metastases on imaging and (2) three consecutive instances of elevated prostate specific antigen (PSA) levels (greater than 2 ng/mL), with the follow-up endpoint set on 28 February 2024.

### 2.2. Extraction of EVs

The extraction steps for plasma extracellular vesicles (p-EVs) were as follows. Firstly, ethylene diamine tetraacetic acid anticoagulated blood samples collected under fasting conditions were centrifuged at 3000 × *g* for 10 min at room temperature within 4 h to separate the plasma. Subsequently, the supernatant was collected after centrifugation at 10,000 × *g* for 10 min at room temperature, aliquoted and stored at −80 °C. One millilitre of plasma was thawed at 4 °C, and EV extraction was performed using the total exosome precipitate reagent (Thermo Fisher, USA) according to the manufacturer’s instructions. In brief, we added 500 μL of phosphate belanced solution (PBS) and 300 μL of precipitant to the plasma, mixed well and incubated at 4 °C for 30 min. Subsequently, the mixture was centrifuged at 12,000 × *g* for 30 min at 4 °C to obtain EV precipitate, and the supernatant was used for cfDNA extraction. For tissue extracellular vesicle (t-EV) extraction, freshly obtained tumour tissue samples were frozen and sectioned. The sections were then digested at 37 °C for 20 min using collagenase IV, protease and DNase I. Subsequently, the digested solution was subjected to gradient centrifugation at 4 °C: 3000 × *g* for 10 min, 2000 × *g* for 10 min and 10,000 × *g* for 30 min [23]. Finally, the supernatant was centrifuged at 110,000 × *g* for 70 min (Beckman Coulter, USA, Type 70 Ti Rotor) to obtain the t-EV precipitate.

### 2.3. Characteristic analysis of EVs

Extracted EVs were identified according to the International Society for Extracellular Vesicles (ISEV) guidelines [24]. Firstly, nanoparticle tracking analysis (NTA) was performed using a ZetaView instrument (Particle Metrix, Germany) to measure the size distribution and concentration of EVs. Then, representative EV morphology was captured using transmission electron microscopy (TEM) (Tecnai, USA). Finally, as previously described [19], western blot (WB) detection was performed on the extracted EV samples using three EV-positive markers and one EV-negative marker: CD9 (Abcam ab20597, 1:3000), HSP70 (Abcam ab18169, 1:1000), TSG101 (Abcam ab12511, 1:200) and calnexin (Abcam ab133615, 1:1000).

### 2.4. DNA extraction and quality control

According to the manufacturer’s instructions, the DNA extraction of extracted EV precipitate using a HiPure Circulating DNA Micro Kit (Magen, China), and the supernatant after p-EV extraction underwent p-cfDNA extraction using a HiPure Circulating DNA Midi Kit (Magen, China). Tissue samples were processed for tissue genomic DNA (t-gDNA) extraction using the HiPure Tissue DNA Mini Kit (Magen, China). DNA concentration was measured using a Qubit 2.0 (Thermo Fisher, USA) to ensure that a sufficient quantity of high-quality DNA was obtained. Fragment size analysis of the extracted p-cfDNA and p-EV DNA was performed using the Agilent High Sensitivity DNA kit (Agilent, USA), followed by analysis with the Agilent 2100 Bioanalyzer according to the manufacturer’s instructions.

### 2.5. Pan-cancer gene targeted sequencing

Targeted sequencing of 571 pan-oncology genes was performed on paired t-gDNA, tissue extracellular vesicle DNA (t-EV DNA), p-EV DNA and p-cfDNA from five patients with PCa. Briefly, the qualified DNA of clinical samples from patients was purified, and adapters were ligated to both ends of the fragments. Next, the extracted DNA was amplified using ligation-mediated polymerase chain reaction (LM-PCR), purified and hybridized to the NanOnco Plus Panel (Nanodigmbio, China) for enrichment. The non-hybridized fragments were then washed out. Both non-captured and captured LM-PCR products were subjected to real-time PCR to estimate the magnitude of enrichment. Subsequently, each captured library was loaded onto a MGISEQ-2000 (MGI Tech, China) platform, and the sequences of each individual were generated as 150 bp paired-end reads. The adapter sequence in the raw data was removed, and high-quality reads were gapped and aligned to the National Center for Biotechnology Information (NCBI) human reference genome (hg19) using BWA with default parameters. Somatic substitutions were detected by MuTect based on BWA alignment, and high-confidence somatic single nucleotide variants were called using the following steps: (1) candidate somatic indels were predicted using the GATK Somatic Indel Detector with default parameters and (2) high-confidence somatic indels were defined after filtering germline events. All high-confidence somatic mutations were filtered using the dbSNP site, which is commonly polymorphic and has no known medical impact [25]. The remaining mutations were annotated using ANNOVAR and were subjected to subsequent analyses.

### 2.6. AR mutation detection

AR hotspot mutations were detected using multiplex-PCR and deep sequencing. Briefly, a primer pool (Supplementary Table 1) containing the AR hotspot mutation target region was designed and synthesized. The first round of PCR was performed to amplify and enrich the target region. Subsequently, the amplicon product was purified using AMPure XP beads, and a second round of PCR was conducted using the first round of PCR products as templates to obtain a library with sequenced molecular tags. The final amplicon product was purified again using AMPure XP beads, and paired-end sequencing of the library was performed using a HiSeq XTen sequencer (Illumina, San Diego, CA, USA). Raw reads were filtered according to two steps. (1) Removing adapter sequence if reads contained them, done by cutadapt (version 1.2.1). (2) Removing low-quality bases from the 3′ to 5′ ends of the reads (*Q* < 20) using PRINSEQ-lite (v 0.20.3). The remaining clean data were then mapped to the NCBI human reference genome (hg19) using BWA with default parameters. Samtools (version 0.1.18) was used to calculate each genotype of the target site. ANNOVAR (2018-04-16) was utilized to detect genetic variants.

### 2.7. Statistical analysis

Data analysis was conducted using GraphPad Prism software (version 9.5.1) or Origin (version 8.5). Comparisons were performed using *t*-tests or one-way analysis of variance where appropriate. Survival analysis for the progression-free survival of patients was conducted using the log-rank test (Kaplan–Meier method). A *P*-value <0.05 was considered statistically significant (*P < 0.05; **P < 0.01; ***P < 0.001).

## 3. Results

### 3.1. Patient Information

**Figure 1.**
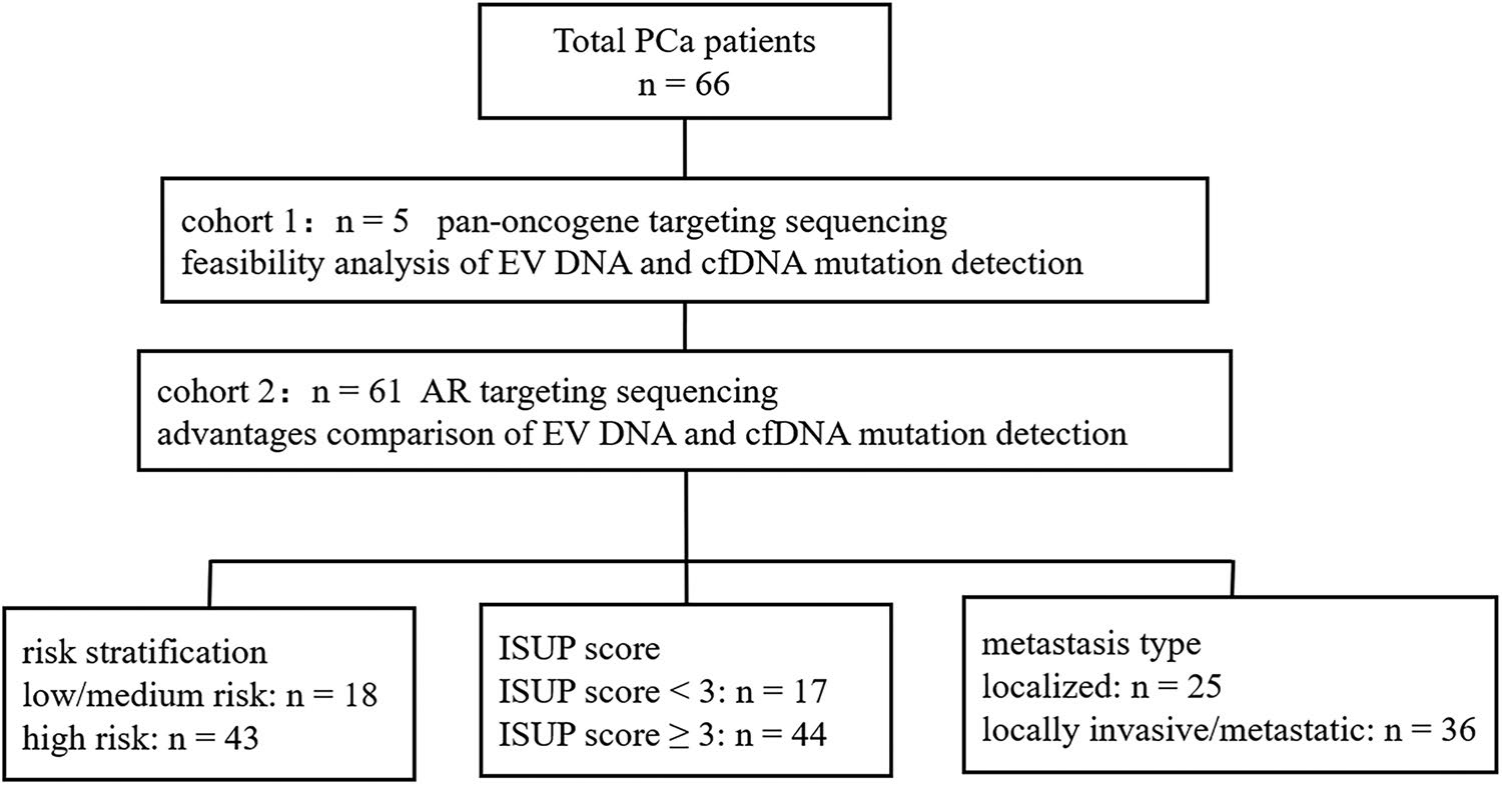
Flow diagram depicting the patients included in this study. Abbreviations: PCa: Prostate cancer; AR: Androgen receptor; EV DNA: Extracellular vesicle DNA; cfDNA: Cell-free DNA; ISUP: The International Society of Urological Pathology.

This study was approved by the Medical Ethics Committee of the Fourth Military Medical University Affiliated Xijing Hospital (No. KY20222066-C-1). A total of 66 patients with PCa confirmed by histopathology were included. Detailed clinical information of the patients is listed in Supplementary Tables 2 and 3. The patients were divided into two cohorts (Figure 1). Cohort 1 included five patients with PCa, from whom paired samples of t-gDNA, t-EV DNA, p-EV DNA and p-cfDNA were collected. Targeted sequencing of pan-cancer genes was performed to explore the feasibility of using p-EV DNA and p-cfDNA as alternatives to tissue samples for the detection of mutations (Supplementary Table 2). Cohort 2 consisted of 61 patients with PCa, of whom 24 had paired tissue samples, while the remaining patients provided only plasma samples. We compared the differences in AR mutation detection in p-EV DNA and p-cfDNA and investigated the relationship between AR mutations and the clinical staging and prognosis of patients with PCa (Supplementary Table 3).

### 3.2. Extraction and identification of EVs

**Figure 2.**
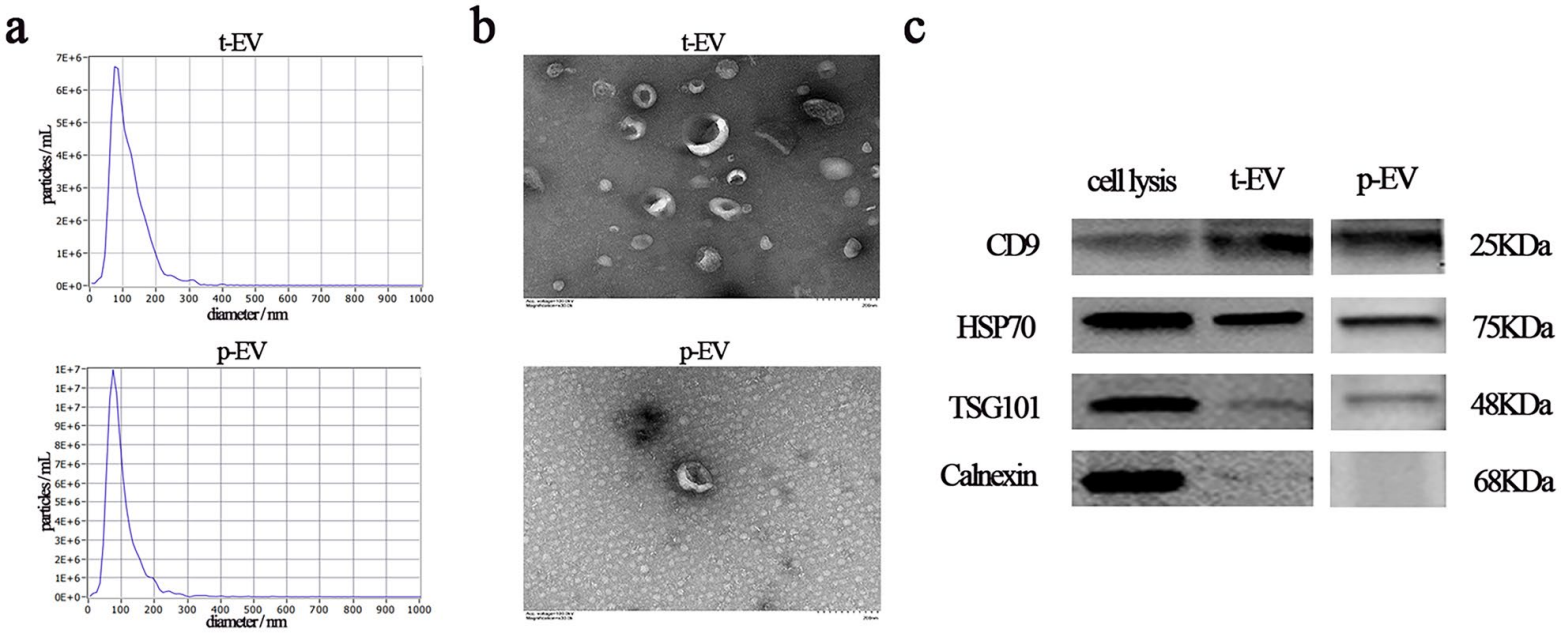
Characterization of EVs: (a) NTA of EVs size distribution; (b) TEM images of EVs and (c) WB analysis of EV protein markers (CD9, HSP70, TSG101) and negative control (calnexin). Abbreviations: NTA: Nanoparticle tracking analysis; TEM: Transmission electron microscope; WB: Western blotting.

We conducted EV extraction and identification in plasma and tissue samples. Firstly, we analysed the size distribution of EVs using NTA, revealing a predominantly 30–150 nm diameter range (Figure 2a). Next, we observed the morphology of EVs under TEM, which displayed a typical ‘cup-shaped’ appearance (Figure 2b). Lastly, we conducted WB to examine three positive markers (CD9, TSG101 and HSP70) and one negative marker (calnexin) in the EVs. The results confirmed that the extracted samples exhibited the molecular characteristics of EVs (Figure 2c). These findings validated the successful extraction of EVs and established the groundwork for subsequent experiments.

### 3.3. Biological characterization of p-EV DNA and cfDNA

**Figure 3.**
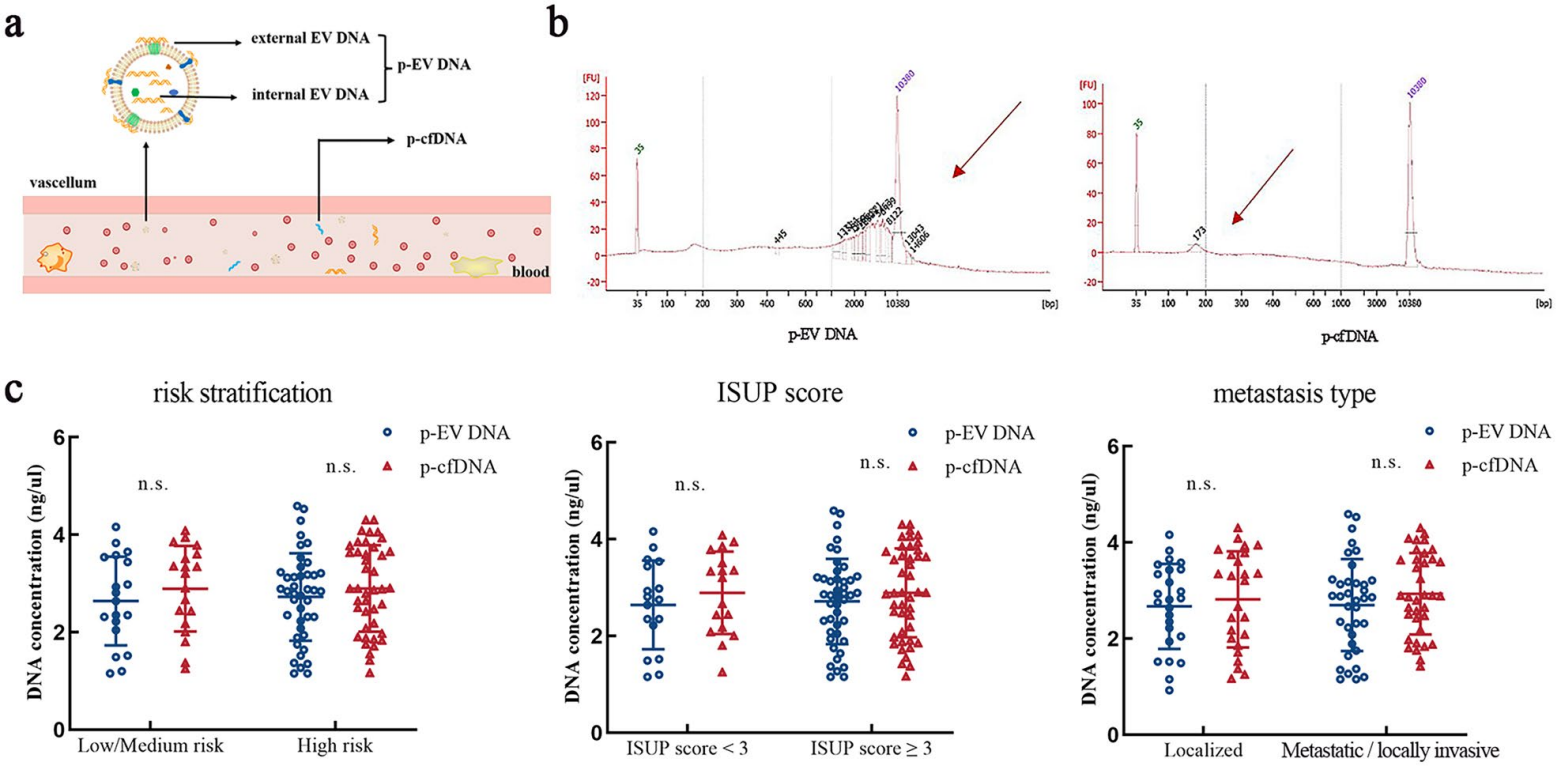
Comparison of biological characteristics of plasma p-EV DNA and p-cfDNA. (a) Schematic diagram of the composition of total plasma DNA. (b) Determination of p-EV DNA and p-cfDNA fragment sizes by capillary electrophoresis. (c) Comparison of plasma p-EV DNA and p-cfDNA concentrations across subgroups. Abbreviations: p-EV DNA: Plasma extracellular vesicle DNA; p-cfDNA: Plasma cell-free DNA.

Total plasma DNA consists of two components: p-EV DNA and p-cfDNA (Figure 3a). These two components were separately extracted. Upon examining the fragment sizes, we observed that p-EV DNA fragments were more dispersed (with a wider range), mostly exceeding 1 kb, whereas p-cfDNA comprised small fragments of approximately 200 bp (Figure 3b). This observation is consistent with the current literature [26,27]. No significant differences were observed in the concentrations of p-EV DNA and p-cfDNA between the different groups of patients with prostate cancer (Figure 3c).

### 3.4. Feasibility verification of mutation detection using p-EV DNA and cfDNA

**Figure 4.**
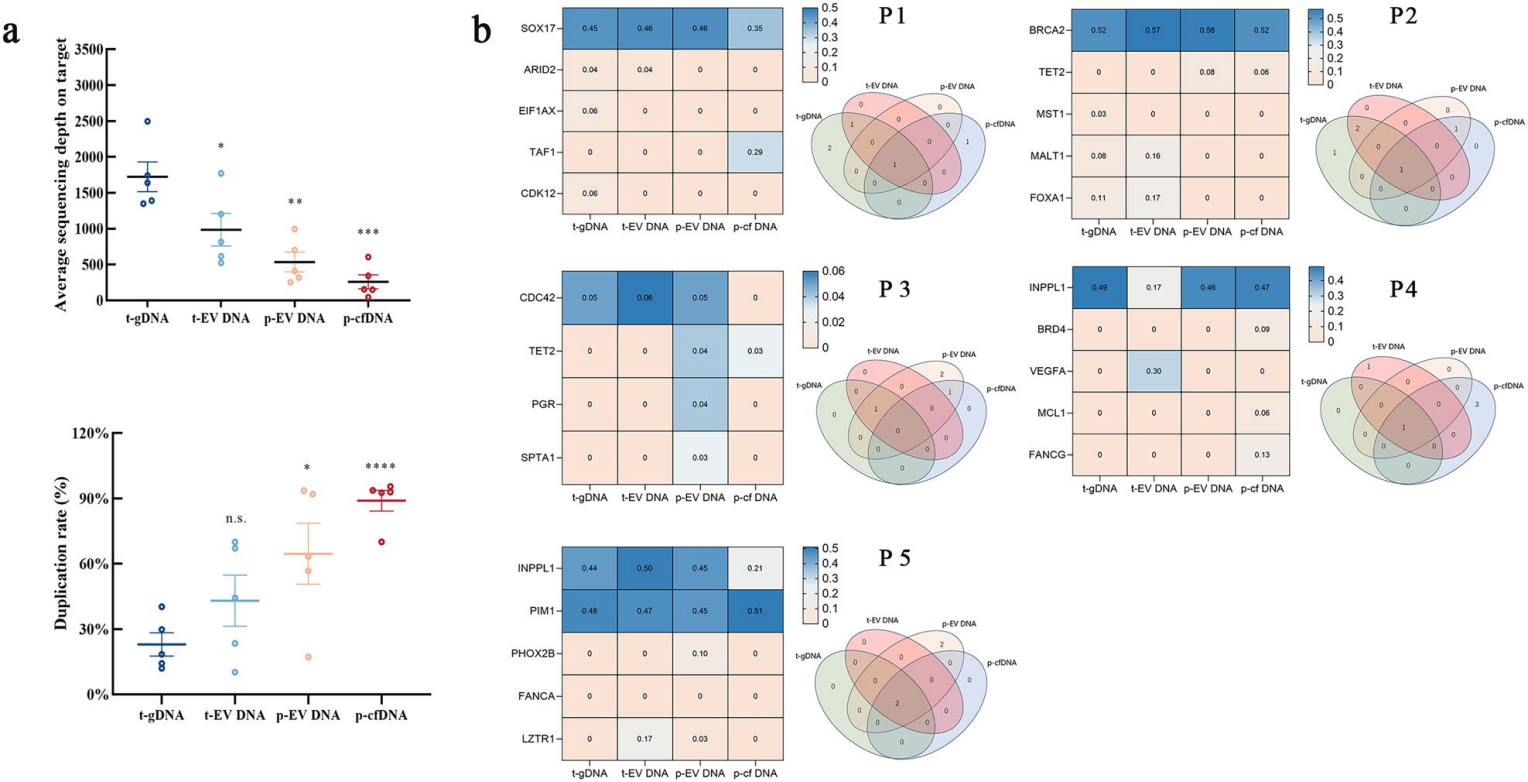
Feasibility analysis of EV DNA and cfDNA mutation detection in cohort 1. (a) Pan-oncogene-targeted sequencing depth and duplication rate of t-gDNA, t-EV DNA, p-EV DNA and p-cfDNA. (b) Heatmap and Venn diagram of the pan-oncogene-targeted sequencing results for t-gDNA, t-EV DNA, p-EV DNA and p-cfDNA from five patients with PCa. Abbreviations: EV DNA: Extracellular vesicle DNA; cfDNA: Cell-free DNA; t-gDNA: Tissue genomic DNA; t-EV DNA: Tissue extracellular vesicle DNA; p-EV DNA: Plasma extracellular vesicle DNA; p-cfDNA: Plasma cell-free DNA.

Using targeted sequencing of pan-cancer genes, we performed mutation detection on paired t-gDNA, t-EV DNA, p-EV DNA and p-cfDNA samples from five patients in cohort 1. As depicted in Figure 4a, with the same DNA input (20 ng), the sequencing depth and duplication rate of the tissue-derived samples were superior to those of the plasma-derived samples, with slightly better results observed for p-EV DNA than for p-cfDNA. In terms of consistency in mutation detection, good concordance was observed between tissue samples (t-gDNA and t-EV DNA). Major mutations associated with PCa detected in tissue samples were also detected in blood samples (p-EV DNA and p-cfDNA), indicating that the use of p-EV DNA and p-cfDNA for mutation detection is feasible. Additionally, blood samples revealed additional mutations that were not detected in the tissue samples. In PCa 3 and PCa 5, p-EV DNA detected more mutations, whereas in PCa 1 and PCa 4, p-cfDNA detected a greater number of mutations. For mutations consistent with tissue, the mutation frequency detected by p-EV DNA was higher than that detected by p-cfDNA in most patients (P1-P4) (Figure 4b).

### 3.5. Comparison of AR mutation detection advantages between p-EV DNA and p-cfDNA

**Figure 5.**
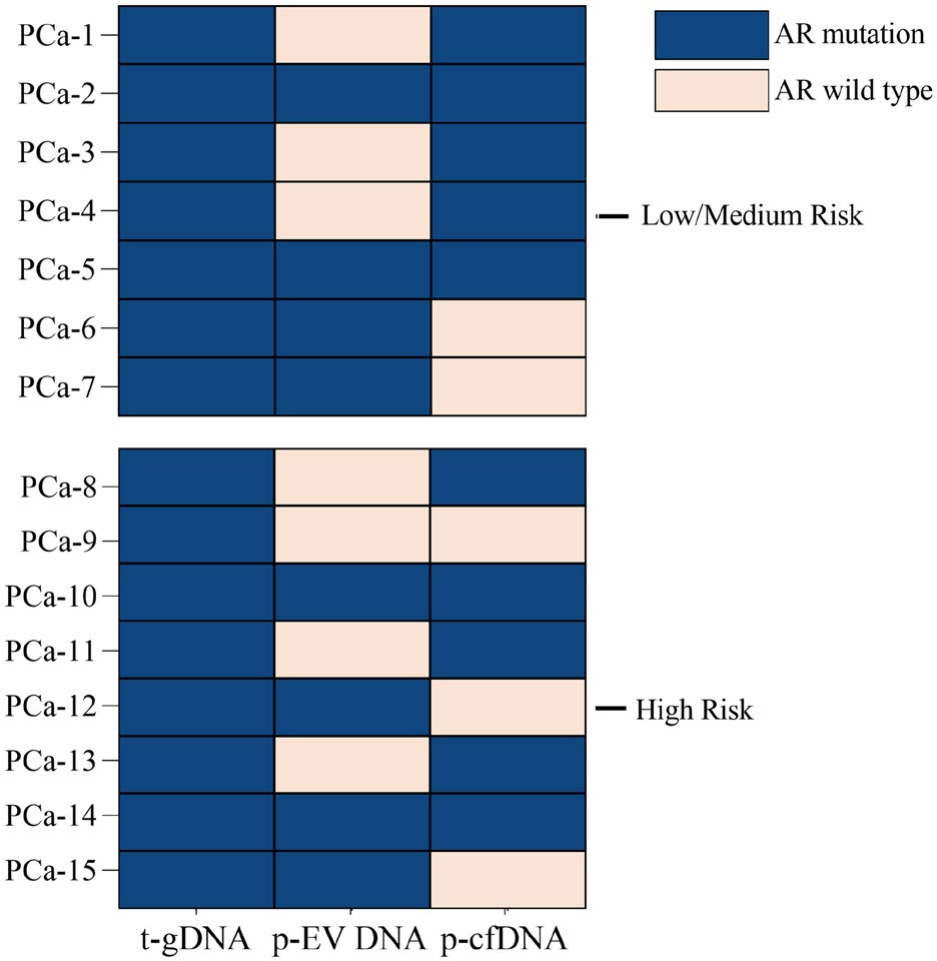
Advantages comparison of EV DNA and cfDNA in AR mutation detection. AR mutation detection results in risk stratification group, blue for AR mutation, pink for AR wild type. Abbreviations: PCa: Prostate cancer; AR: Androgen receptor; EV DNA: Extracellular vesicle DNA; cfDNA: Cell-free DNA; t-gDNA: Tissue genomic DNA; p-EV DNA: Plasma extracellular vesicle DNA; p-cfDNA: Plasma cell-free DNA.

AR mutations are one of the primary pathways leading to ARPI resistance and are closely associated with poor prognosis in patients with PCa. To further determine which component, p-EV DNA or p-cfDNA, has the advantage of mutation detection, we compared the detection of AR mutations in paired p-EV DNA and p-cfDNA samples from fifteen patients known to harbour AR hotspot mutations based on tissue testing (Figure 5). Among the fifteen patients, seven were categorized as having low to intermediate risk, while eight were classified as high risk. In the low-to-intermediate-risk group, mutations were detected in p-EV DNA samples from four patients and in p-cfDNA samples from five patients. Among them, only two patients had the same mutation in both p-EV DNA and p-cfDNA, with a higher mutation frequency detected in p-cfDNA samples. No disease progression occurred during follow-up in these patients. Among the eight high-risk patients, mutations were detected in p-EV DNA samples from four patients, all of whom experienced disease progression. While mutations were detected in p-cfDNA samples from five patients, three showed no disease progression during the follow-up period. Two patients had mutations in both types of samples, and both had disease progression. However, a higher mutation frequency was detected in the p-cfDNA sample. cfDNA samples appeared to be more sensitive in detecting AR mutations, but EV DNA mutation results were associated with poor patient outcomes.

### 3.6. AR mutations in p-EV DNA are more associated with poor prognosis in patients with PCa

To further compare the differences between p-EV DNA and p-cfDNA in AR mutation detection and explore the relationship between AR mutations in plasma and different clinical stages and prognosis of PCa, we conducted AR mutation detection on paired p-EV DNA and p-cfDNA samples from 61 patients with PCa. The results revealed that a larger proportion of patients with higher malignancy levels of PCa (high risk, ISUP score ≥3, metastatic PCa) had AR mutations. In addition, the frequency of mutation detection in p-EV DNA was generally slightly lower than that in p-cfDNA (Table 1).

**Table 1.** AR mutation call rates.

Way of grouping		p-EV DNA call rate (%)	p-cfDNA call rate (%)	Total mutation call rate (%)
Risk stratification	Low/medium risk	5/18 (27.78)	7/18 (38.89)	10/18 (55.56)
	High risk	16/43 (37.21)	20/43 (46.51)	25/43 (58.14)
ISUP score	ISUP score < 3	4/17 (23.53)	6/17 (35.29)	9/17 (52.94)
	ISUP score ≥ 3	17/44 (38.64)	21/44 (47.73)	26/44 (59.09)
Metastasis type	Localized	9/25 (36.00)	10/25 (40.00)	13/25 (52.00)
	Locally invasive/metastatic	12/36 (33.33)	17/36 (47.22)	22/36 (61.11)

Abbreviations: AR: Androgen receptor; ISUP: The International Society of Urological Pathology; p-EV DNA: Plasma extracellular vesicle DNA; p-cfDNA: Plasma cell-free DNA.

**Figure 6.**
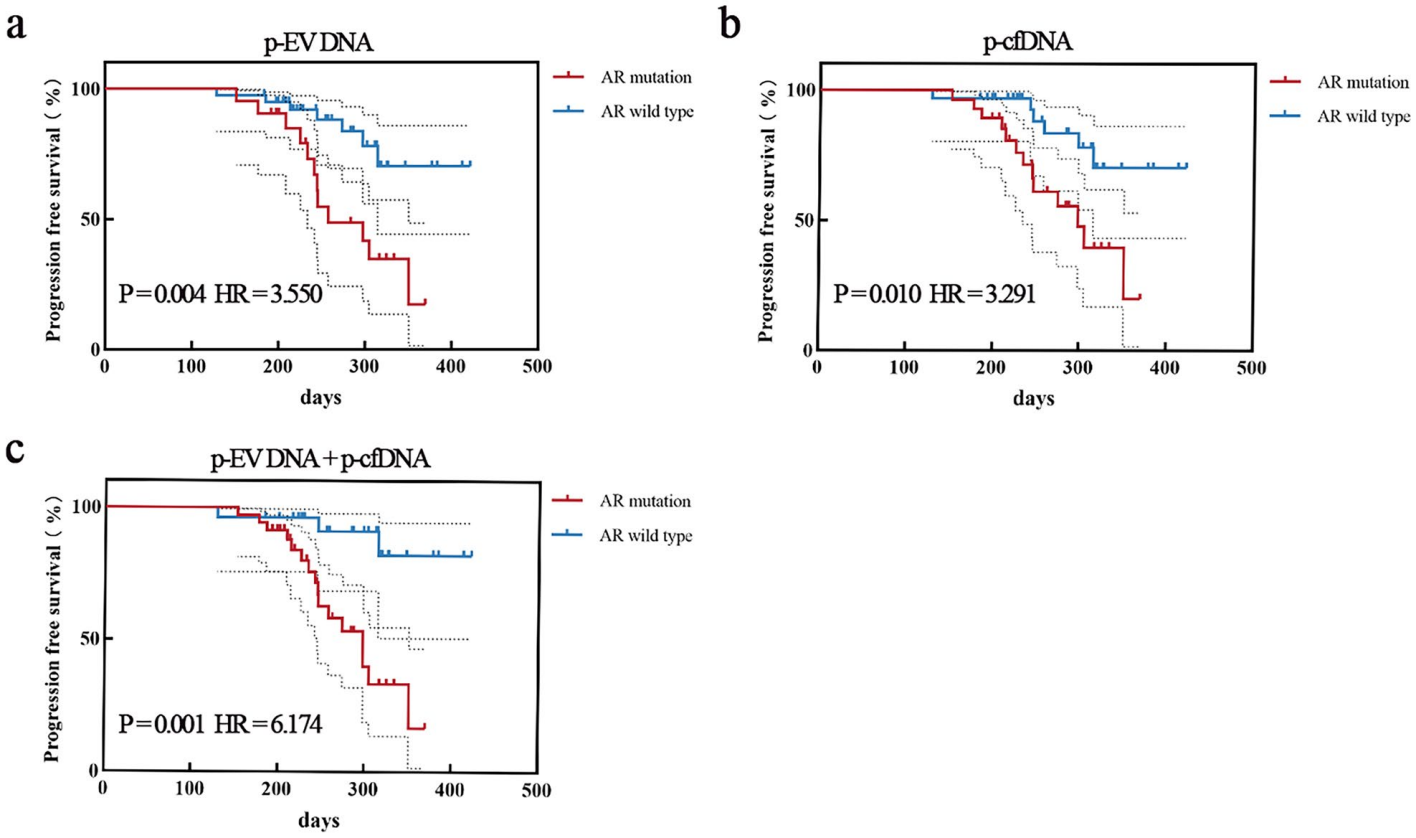
Association between AR mutations and patient clinical outcomes. (a) Kaplan–Meier curves for progression-free survival (PFS) in patients with and without AR mutations in p-EV DNA (*n* = 61*, P* = 0.004, HR = 3.550). (b) Kaplan–Meier curves for PFS in patients with and without AR mutations in p-cfDNA (*n* = 61*, P* = 0.010, HR = 3.291). (c) Kaplan–Meier curves for PFS in patients with and without AR mutations in total plasma DNA (p-EV DNA and p-cfDNA) (*n* = 61*, P* = 0.001, HR = 6.174). Abbreviations: AR: Androgen receptor; p-EV DNA: Plasma extracellular vesicle DNA; p-cfDNA: Plasma cell-free DNA; HR: Hazard ratio.

Combined with the follow-up results, it was observed that among the seven patients with mutations detected in p-EV DNA but not in p-cfDNA, three patients (42%) experienced disease progression (radiographic or PSA progression) during subsequent follow-up. Among the 14 patients with mutations detected in p-cfDNA but not in p-EV DNA, only four (29%) experienced disease progression during subsequent follow-up. If the results of the two are analysed together, among the 14 patients who had mutations detected in both samples (p-EV DNA and p-cfDNA), nine patients (64%) experienced disease progression during follow-up. Only three (11%) of the 26 patients who had no mutations in either sample ultimately had disease progression. Survival analysis results suggested that patients with detected AR mutations had a higher risk of mortality than those without detected mutations, especially in p-EV DNA samples. Moreover, when the mutation detection results of the two sample types were combined, intergroup differences became more significant (Figure 6).

## 4. Discussion

As one of the three major targets of liquid biopsy, cfDNA is widely used in precision diagnosis and treatment because of its convenient acquisition and ability to overcome the heterogeneity of tissue biopsy. However, there are still many problems with blood cfDNA detection: (1) cfDNA is highly fragmented (approximately 200 bp), with a half-life of only approximately 2 h, which is inconvenient for clinical operations [11,15,28]; (2) blood cfDNA contains a large number of components derived from non-tumour cells, making it challenging to identify tumour-specific changes from a highly abundant normal background, especially in the early stages of tumours [14] and (3) cfDNA mainly originates from necrotic and apoptotic cells, characteristic of advanced diseases and may not accurately reflect the state of living cells [29]. EV DNA has emerged as a new liquid biopsy target and is expected to overcome these problems. The advantages of EV DNA are as follows: (1) EV DNA fragments are longer and more stable [30]; (2) EV are isolated from tumours using the immunoaffinity method, facilitating the acquisition of tumour-specific EV DNA [17,31] and (3) EV are mainly secreted by living cells, suggesting that EV DNA may provide a better and earlier reflection of the tumour status [28].

Previous studies have preliminarily explored the value of EV DNA as a tumour marker and found that it has certain advantages. (1) EV DNA is more sensitive: in a study related to epidermal growth factor receptor (EGFR) genotyping of non-small cell lung cancer, the authors simultaneously detected EGFR mutations using EV DNA and cfDNA discovering that EV DNA samples exhibited greater sensitivity [32]. Additionally, two studies found that tumour-associated mutations were more enriched in EV DNA, particularly in early-stage tumours [18,19]. (2) EV DNA shows better consistency with tissue biopsy: some researchers conducted mutation detection using cfDNA and EV DNA in the supernatant of pleural effusion and found that EV DNA demonstrated a higher consistency with tissue biopsy [33]. (3) EV DNA is better for prognostic judgement: in a related study on the prognosis of patients with pancreatic cancer based on KRAS mutation, EV DNA samples were better than ctDNA samples for prognostic judgement [34]. In our study, we found that although cfDNA samples could detect more AR mutations than EV DNA, the AR mutations detected by EV DNA were more closely associated with the poor prognosis of patients. This finding confirms the advantages of EV DNA as a diagnostic marker of disease to some extent.

Currently, several research teams have detected AR variation through cfDNA in patients with PCa and confirmed their association with poor patient prognosis [12,13]. However, there is a lack of research on EV DNA in prostate cancer. Only Vagner et al. [27] and Lázaro-Ibáñez et al. [35] have conducted preliminary explorations, confirming that DNA in EVs reflects genetic aberrations in the cell of origin. However, these studies primarily utilized cell culture supernatants and included only a few clinical samples. Therefore, this study aimed to explore the value of EV DNA as a diagnostic target for PCa. Initially, we compared the biological characteristics of EV DNA and cfDNA. Consistent with previous reports, the EV DNA fragment size was larger than that of cfDNA [30]. However, there was no significant difference in the concentrations of EV DNA and cfDNA among the different patients, which differs from the conclusion of Klump et al. [36] that the concentration of EV DNA was higher. We speculate that this may have been caused by the different separation and extraction methods. Klump et al. also filtered the supernatant after EV separation before cfDNA extraction, which may have resulted in cfDNA loss. To explore the feasibility of mutation detection using EV DNA and cfDNA, we conducted mutation detection in paired tissue and plasma samples. We found that major PCa-related mutations detected in tissues were also detectable in blood samples, suggesting that mutation detection using p-EV DNA and cfDNA is feasible. However, owing to the high cost of multigene targeted sequencing, we selected a gene with a high mutation frequency in PCa as the research focus. AR has a high mutation frequency in PCa, especially in advanced patients, and is closely associated with resistance to various AR-targeted drugs and poor patient prognosis [8,9]. Therefore, we focused on the detection of AR mutations in a follow-up study. To further compare the differences in mutation detection between EV DNA and cfDNA, we conducted AR mutation detection in paired EV DNA and cfDNA from patients with confirmed AR hotspot mutations. Although more mutations were detected in cfDNA than in EV DNA, the mutations detected in EV DNA showed a stronger correlation with tumour prognosis. This trend was also observed in the subsequently expanded cohort. These findings suggest that AR mutations in EV DNA may better correlate with poor prognosis in PCa than those in cfDNA.

However, our study has certain limitations. Firstly, our cohort was small, and further evidence of the advantages of the two needs to be verified using a larger sample size. Secondly, due to the high cost associated with pan-cancer gene targeted sequencing (thousands of RMB per sample), in contrast to the comparatively lower cost of detecting hotspot mutations of single gene using multiplex-PCR and deep sequencing (only hundreds RMB per sample), investigating AR mutations was undertaken as the primary focus of our expanded sample experiment. Consequently, we were unable to compare the mutation detection efficiency of EV DNA and cfDNA at the whole genomic level. Additionally, apart from mutations, AR copy number variation and spliceosome variation can also cause ARPI resistance; therefore, focusing solely on AR mutations will have certain limitations in the prognosis analysis of PCa. Moreover, AR has a higher mutation frequency in patients with advanced PCa, and studying AR mutations alone cannot fully reflect the advantages of EV DNA in early-stage tumours. Lastly, this was only a preliminary exploratory study, and the follow-up duration was short. To further explore the role of EV DNA and cfDNA AR mutation detection in PCa prognosis, we will continue to monitor the disease conditions of the enrolled patients.

In summary, our study systematically compared EV DNA and cfDNA in terms of biological characteristics, feasibility of mutation detection, sensitivity of mutation detection and prognostic value of the detected mutations. We explored the potential of EV DNA and cfDNA as diagnostic markers for PCa and evaluated the relationship between plasma AR mutations and PCa staging and prognosis. Our results provide an important basis for further research on the significance of the AR gene in clinical monitoring of PCa and the potential of EV DNA as a new tumour marker.

## Supplementary Material

Supplemental Material

## Data Availability

The data presented in this study are available on request from the corresponding authors.
