# Supplementary material for: Comparison of androgen receptor mutation detection between plasma extracellular vesicle DNA and cell-free DNA and its relationship to prostate cancer prognosis
Source: Ann Med. 2024 Nov 13;56(1):2426770. doi: 10.1080/07853890.2024.2426770 (PMC11562022; doi:10.1080/07853890.2024.2426770)
Supplement: Supplemental Material [file IANN_A_2426770_SM0246.docx]

| **Supplementary Table 1. Regions and primers** | | | | | |
| --- | --- | --- | --- | --- | --- |
| assay | chromosome | start | end | forward primer | reverse primer |
| 1 | X | 66931399 | 66931491 | CCCACTTGACCACGTGTACAAGCTG | CATCTTTCTGAATGTCCTGGAAGCC |
| 2 | X | 66937366 | 66937453 | CCAGGAGCACTTACTCATTGAAAAC | ACGACCAGATGGCTGTCATTCAGTA |
| 3 | X | 66943518 | 66943592 | GATCTCTGCCATCATTTCCGGAAAG | AACCCTGTTTTTCTCCCTCTTATTG |

| **Supplementary Table 2. Clinical information of PCa patiens in cohort 1** | | | | | | | | | | |
| --- | --- | --- | --- | --- | --- | --- | --- | --- | --- | --- |
| Sample ID | Gender | Age | Risk stratification | Gleason score | ISUP score | Metastasis type | PSA value (ng/mL) | TNM staging | | |
|  |  |  |  |  |  |  |  | T | N | M |
| PCa-1 | male | 68 | medium risk | 7 | 2 | localized | 8.52 | 2 | X | 1 |
| PCa-2 | male | 64 | high risk | 9 | 5 | metastatic | 37.47 | 3 | 1 | 0 |
| PCa-3 | male | 74 | high risk | 8 | 4 | localized | 5.16 | 2 | X | 0 |
| PCa-4 | male | 61 | high risk | 7 | 3 | metastatic | 28.67 | 3 | 0 | 0 |
| PCa-5 | male | 60 | medium risk | 7 | 2 | localized | 9.24 | 2 | 0 | 0 |

Abbreviations: PCa: prostate cancer; AR: Androgen receptor; ISUP: the international society of urological pathology; PSA: prostate specific antigen; TNM: tumor node metastasis.

| **Supplementary Table 3. Clinical information of PCa patiens in cohort 2** | | | | | | | | | | | | | | | | | |
| --- | --- | --- | --- | --- | --- | --- | --- | --- | --- | --- | --- | --- | --- | --- | --- | --- | --- |
| Sample ID | Gender | Age | Risk stratification | Gleason score | ISUP score | Metastasis type | PSA value (ng/mL) | TNM staging | | | Disease progression | Progression free survival（Days） | AR Mutation in t-gDNA* | AR Mutation in  p-EV DNA | | AR Mutation* in  p-cfDNA | |
|  |  |  |  |  |  |  |  | T | N | M |  |  |  | mutation site | mutation frequency | mutation site | mutation frequency |
| PCa-1 | male | 60 | medium risk | 7 | 2 | localized | 5.69 | 2 | x | x | no disease progression | 287 | p.F877L | - | - | p.F877L | 0.51 |
| PCa-2 | male | 73 | medium risk | 7 | 3 | localized | 5.65 | 2 | x | x | no disease progression | 317 | p.T878A | p.T878A | 19.7 | p.T878A | 32.9 |
| PCa-3 | male | 70 | medium risk | 7 | 2 | metastatic | 19.5 | - | - | - | no disease progression | 209 | p.F877L | - | - | p.H875Y, p.F877L, p.T878A | 2.6, 2.8, 1.6 |
| PCa-4 | male | 69 | medium risk | 7 | 2 | localized | 4.95 | 2 | x | x | no disease progression | 262 | p.F877L, p.T878A | - | - | p.H875Y, p.F877L, p.T878A | 4.4 ,4.3, 3.14 |
| PCa-5 | male | 69 | low risk | 6 | 1 | localized | 6.85 | 2 | x | x | no disease progression | 284 | p.W702P, p.F877L, p.T878A | p.F877L | 0.57 | p.F877L, p.T878A | 1.27, 0.76 |
| PCa-6 | male | 69 | medium risk | 7 | 2 | localized | 10.34 | 2 | x | x | no disease progression | 201 | p.F877L, p.T878A | p.F877L, p.T878A | 1.09, 0.92 | - | - |
| PCa-7 | male | 74 | low risk | 6 | 1 | localized | 3.9 | - | - | - | no disease progression | 198 | p.F877L | p.W742X | 0.8 | - | - |
| PCa-8 | male | 64 | high risk | 7 | 3 | locally invasive | 18.2 | 2 | x | x | no disease progression | 244 | p.H875R | - | - | p.H875R | 0.94 |
| PCa-9 | male | 45 | high risk | 8 | 4 | metastatic | - | 3b | 1 | x | no disease progression | 184 | p.H875Y | - | - | - | - |
| PCa-10 | male | 58 | high risk | 9 | 5 | localized | 1.54 | 2 | x | x | PSA progression | 226 | p.F877L | p.F877L | 0.4 | p.W702l, p.F877L | 1.34,0.83 |
| PCa-11 | male | 66 | high risk | 9 | 5 | locally invasive | 23.5 | 3b | x | x | no disease progression | 213 | p.T877S | - | - | p.F877S | 0.57 |
| PCa-12 | male | 73 | high risk | 8 | 4 | metastatic | - | 3b | 1 | x | PSA progression | 242 | p.F877L | p.W742R, p.F875Y, p.F877L | 0.55, 1.65, 2.03 | - | - |
| PCa-13 | male | 73 | high risk | 7 | 3 | metastatic | 124.6 | - | - | - | no disease progression | 287 | p.F877L | - | - | p.F877L, p.T878S | 0.53, 0.52 |
| PCa-14 | male | 69 | high risk | 7 | 3 | locally invasive | 7.08 | 2 | x | x | Radiographic progression | 245 | p.F877L, p.T878A | p.T878A | 1.27 | p.F877L | 1.2 |
| PCa-15 | male | 66 | high risk | 9 | 5 | metastatic | 59 | 3b | 1 | x | Radiographic progression | 351 | p.T877A | p.F877L, p.T878A | 1.0, 1.2 |  |  |
| PCa-16 | male | 72 | high risk | 7 | 3 | localized | 12.99 | 2 | x | x | no disease progression | 370 | - | p.T878A | 23.73 | p.T878A | 15.4 |
| PCa-17 | male | 77 | medium risk | 7 | 2 | localized | 6.55 | 2 | x | x | PSA progression | 298 | - | - | - | p.W742C, p.T 878A | 16.52, 0.75 |
| PCa-18 | male | 74 | high risk | 9 | 5 | metastatic | 31.75 | 3b | 1 | x | PSA progression | 274 | - | - | - | p.T878A | 1 |
| PCa-19 | male | 69 | high risk | 8 | 4 | metastatic | 24 | 3b | 1 | x | no disease progression | 226 | - | - | - | - | - |
| PCa-20 | male | 71 | high risk | 8 | 4 | locally invasive | 24.07 | 2 | x | x | no disease progression | 284 | - | - | - | - | - |
| PCa-21 | male | 40 | medium risk | 7 | 2 | localized | 10.67 | 2 | x | x | no disease progression | 317 | - | - | - | - | - |
| PCa-22 | male | 73 | high risk | 9 | 5 | metastatic | 1613 | 3a | 1 | x | no disease progression | 384 | - | - | - | - | - |
| PCa-23 | male | 76 | high risk | 9 | 5 | metastatic | - | 2c | 1 | x | PSA progression | 245 | - | - | - | - | - |
| PCa-24 | male | 74 | high risk | 9 | 5 | locally invasive | - | 2 | x | x | no disease progression | 200 | - | - | - | - | - |
| PCa-25 | male | 73 | high risk | 7 | 3 | localized | 40.17 | 2 | x | x | no disease progression | 422 | no | - | - | - | - |
| PCa-26 | male | 68 | medium risk | 7 | 2 | localized | 12.9 | 2 | x | x | no disease progression | 413 | no | - | - | - | - |
| PCa-27 | male | 59 | high risk | 7 | 3 | metastatic | 68 | - | - | - | no disease progression | 326 | no | - | - | - | - |
| PCa-28 | male | 77 | high risk | 7 | 3 | metastatic | 24.7 | - | - | - | no disease progression | 325 | no | p.T878A | 0.56 | p.F877L, p.T878A | 0.59, 0.77 |
| PCa-29 | male | 77 | medium risk | 7 | 2 | localized | 18.43 | 2 | x | x | no disease progression | 314 | no | - | - | - | - |
| PCa-30 | male | 67 | medium risk | 7 | 2 | localized | 5.69 | 2 | x | x | no disease progression | 298 | no | - | - | - | - |
| PCa-31 | male | 65 | high risk | 7 | 3 | localized | 27.3 | 2 | x | x | Radiographic progression | 209 | no | p.F877L | 0.62 | p.T878A | 1.4 |
| PCa-32 | male | 71 | high risk | 8 | 4 | metastatic | 792.2 | - | - | - | no disease progression | 327 | no | - | - | - | - |
| PCa-33 | male | 69 | high risk | 9 | 5 | metastatic | 1039 | - | - | - | no disease progression | 319 | no | - | - | - | - |
| PCa-34 | male | 70 | high risk | 8 | 4 | metastatic | 49.9 | 3b | 1 | x | PSA progression | 129 | no | - | - | - | - |
| PCa-35 | male | 37 | high risk | 9 | 5 | metastatic | 62.38 | - | - | - | no disease progression | 303 | no | - | - | - | - |
| PCa-36 | male | 65 | high risk | 10 | 5 | metastatic | 100 | - | - | - | PSA progression | 177 | no | p.F877L, p.T878A | 0.8, 5.3 | p.T878A | 10.68 |
| PCa-37 | male | 79 | high risk | 8 | 4 | metastatic | 78 | - | - | - | no disease progression | 186 | no | - | - | p.F877L, p.T878A | 5.89, 17.97 |
| PCa-38 | male | 73 | high risk | 7 | 3 | metastatic | 28.47 | - | - | - | Radiographic progression | 315 | no | - | - | - | - |
| PCa-39 | male | 66 | high risk | 9 | 5 | metastatic | 104.9 | 3b | 1 | x | PSA progression | 234 | no | p.T878A | 1.4 | p.F877L | 0.53 |
| PCa-40 | male | 64 | high risk | 9 | 5 | metastatic | 579.3 | 3b | 1 | x | no disease progression | 206 | no | - | - | p.W742C | 3.28 |
| PCa-41 | male | 59 | high risk | 8 | 4 | metastatic | 431.1 | 3b | 1 | x | Radiographic progression | 258 | no | p.F877L | 0.51 | - | - |
| PCa-42 | male | 71 | high risk | 9 | 5 | localized | 1002 | 2 | 0 | x | no disease progression | 312 | no | - | - | - | - |
| PCa-43 | male | 37 | high risk | 9 | 5 | metastatic | 62.38 | 3b | 1 | x | no disease progression | 218 | no | - | - | p.F877L, T878A | 0.56, 1.5 |
| PCa-44 | male | 65 | high risk | 9 | 5 | metastatic | 20.63 | - | - | - | no disease progression | 198 | no | - | - | p.F877L, T878A | 0.67, 1.7 |
| PCa-45 | male | 70 | high risk | 7 | 3 | localized | 21 | 2 | x | x | no disease progression | 347 | no | - | - | - | - |
| PCa-46 | male | 67 | high risk | 9 | 5 | metastatic | 102 | - | - | - | no disease progression | 334 | no | p.W742L | 0.81 | p.T878A | 0.63 |
| PCa-47 | male | 69 | high risk | 8 | 4 | metastatic | 170 | - | - | - | no disease progression | 232 | no | p.F877L | 0.51 | - | - |
| PCa-48 | male | 73 | high risk | 9 | 5 | metastatic | 36.17 | 4 | 0 | 1b | no disease progression | 255 | no | - | - | - | - |
| PCa-49 | male | 81 | high risk | 10 | 5 | metastatic | 84.19 | - | - | - | PSA progression | 152 | no | p.H875Y, p.T878A | 0.65 0.5 | T878A | 8.1 |
| PCa-50 | male | 77 | high risk | 7 | 3 | localized | 54 | - | - | - | no disease progression | 378 | no | - | - | - | - |
| PCa-51 | male | 63 | low risk | 6 | 1 | localized | 4.95 | 2 | x | x | no disease progression | 286 | no | - | - | - | - |
| PCa-52 | male | 78 | medium risk | 7 | 2 | localized | 24.7 | - | - | - | no disease progression | 229 | no | - | - | - | - |
| PCa-53 | male | 65 | medium risk | 7 | 2 | localized | 118.9 | 2 | x | x | no disease progression | 222 | no | - | - | - | - |
| PCa-54 | male | 75 | low risk | 6 | 1 | localized | 8.38 | - | - | - | no disease progression | 216 | no | - | - | - | - |
| PCa-55 | male | 72 | medium risk | 7 | 2 | localized | 13.03 | 2 | x | x | PSA progression | 214 | no | - | - | p.H875Y, p.F877L | 1.62, 1.89 |
| PCa-56 | male | 73 | medium risk | 7 | 2 | localized | 7.23 | - | - | - | no disease progression | 192 | no | p.F877Y | 0.52 | - | - |
| PCa-57 | male | 69 | high risk | 7 | 3 | localized | 6.22 | - | - | - | PSA progression | 305 | no | p.F877Y | 0.54 | p.H875Q | 0.79 |
| PCa-58 | male | 51 | high risk | 9 | 5 | metastatic | 27.09 | 3b | 1 | x | PSA progression | 246 | no | p.F877L, p.T878A | 1.12, 1.03 | p.W702P, p.T878A | 1.71, 0.56 |
| PCa-59 | male | 62 | high risk | 8 | 4 | metastatic | 100 | - | - | - | no disease progression | 258 | no | - | - | - | - |
| PCa-60 | male | 55 | high risk | 9 | 5 | metastatic | 345.5 | - | - | - | PSA progression | 186 | no | - | - | p.F877S | 1.33 |
| PCa-61 | male | 76 | high risk | 7 | 3 | metastatic | 44.07 | - | - | - | PSA progression | 298 | no | p.W742X | 0.93 | - | - |

*: "-" means no AR mutation is detected; "no"means no tissue sample was obtained for AR mutation detection.

Abbreviations: PCa: prostate cancer; AR: Androgen receptor; ISUP: the international society of urological pathology; PSA: prostate specific antigen; TNM: tumor node metastasis; t-gDNA: tissue genomic DNA; p-EV DNA: plasma extracellular vesicle DNA; p-cfDNA: plasma cell-free DNA.
